# A novel online Food Recall Checklist for use in an undergraduate student population: a comparison with diet diaries

**DOI:** 10.1186/1475-2891-8-13

**Published:** 2009-02-19

**Authors:** Fiona Comrie, Lindsey F Masson, Geraldine McNeill

**Affiliations:** 1 Institute of Applied Health Sciences, University of Aberdeen, Foresterhill, Aberdeen, AB25 2ZD, UK

## Abstract

**Background:**

University students are commonly overlooked when diet of populations is measured and there is a lack of comprehensive dietary assessment in whole university student populations. To measure diet of undergraduate students, a new online 121-item Food Recall Checklist (FoRC) was designed as an alternative to a non-weighed record (food diary). This article reports the comparison between the new dietary assessment method (FoRC) and the food diary as a measure of energy (kJ), fat (g), Non-Starch Polysaccharide (NSP) (g), fruit and vegetables (g), breakfast cereal (g) and bread (g) and alcohol (units) intake.

**Methods:**

Fifty-three students at the University of Aberdeen completed four days of FoRC then four days food diary. Median agreement and correlation between the two methods was assessed for foods and nutrients using the Spearman's rank correlation co-efficient and the Wilcoxon signed ranks test. Agreement between FoRC and food diary was assessed using the Bland-Altman method.

**Results:**

The mean time taken to complete FoRC for one day was 7.4 minutes. Intakes of fat (g and % food energy), NSP and bread were similar between FoRC and the food diary. Median energy intake was 8185 kJ in the food diary and 8007 kJ in FoRC. However, FoRC recorded significantly lower intakes of energy and alcohol and significantly higher intakes of fruit and vegetables and breakfast cereal compared with the food diary. There was considerable variation in agreement between methods at the individual level. For all variables except alcohol and percentage energy from fat, correlation co-efficients were statistically significant and greater than 0.5.

**Conclusion:**

At the group level, four days of FoRC showed good median agreement with the food diary and there was high correlation between methods for most foods and nutrients. This suggests that this novel method of assessing diet can provide a useful alternative for assessing group mean intakes but that individual intakes may need to be interpreted with care.

## Background

University students are commonly overlooked when diet of populations is measured and there is a lack of comprehensive dietary assessment in university student populations. These individuals are less likely to be included in family, workplace or community-based diet and health interventions. The transition to catering for oneself can be a period of changing dietary habits [1]. There is evidence that chronic disease risk factors may be established in youth and can persist into adulthood [2,3], it is therefore important to monitor diet in the university population. With an accurate measure of diet intake, dietary feedback may be provided to participants and this may promote healthy diet change in a population [4]. Since a student population may not prioritise eating healthily above other social and academic commitments [5], providing diet feedback and advice may be useful.

In studies of diet, typical assessment methods are Food Frequency Questionnaires (FFQ) or weighed food records. While these measures have been shown to provide valid estimates of diet, both have limitations in terms of acceptability to participants and time taken to interpret the data. Ideally, a new dietary assessment method for use in a university population would provide a good estimate of habitual diet intake, would be acceptable to participants and would have low costs in terms of money and time. There is evidence of a lower response rate in health and diet surveys amongst young adults, especially males [6,7], so any new method should appeal to these respondents.

Measuring diet in the hard-to-reach university population is an issue which can be bridged using newer technologies in dietary assessment. There are a number of new ways to measure diet and to access health information, including mobile phones, digital cameras and palmtop computers, as well as other computer-based methods [8-12]. In Great Britain, 90% of individuals aged 16–24 years will have accessed the Internet in the previous three months [13] and it is now commonplace for institutions such as universities to conduct much of their administration via the Internet. This growth in Internet access has made collecting dietary data online much easier. Data collected in this fashion can be entered remotely and electronically by participants and returned pre-coded for analysis.

To collect retrospective dietary data from undergraduate students, a new, online 121-item, 24-h Food Recall Checklist (FoRC) was developed. FoRC was designed to estimate intake of energy (kJ), fat (g), non-starch polysaccharide (NSP) (g), fruit and vegetables (g), breakfast cereal (g) and bread (g) and alcohol (units) on the previous day. These nutrients and foods were chosen from the UK Dietary Reference Values [14] and Scottish Dietary Targets [15] as important targets to monitor for public health. Pilot work on FoRC in another sample of students (data not presented) had shown that participants found the instructions easy to understand and that most participants could easily locate the foods they had consumed within the questionnaire.

The aim of the comparison study was to assess whether four days of FoRC could be used to give an estimate of diet intake in place of four days of a more traditional method of dietary assessment. This article reports the comparison between the new dietary assessment method (FoRC) and the non-weighed record as a measure of energy (kJ), fat (g), Non-Starch Polysaccharide (NSP) (g), fruit and vegetables (g), breakfast cereal (g) and bread (g) and alcohol (units) intake. The non-weighed diet record was chosen as it was easier to complete than a weighed record and it was not subject to the same errors of recall as FoRC. The non-weighed diet record has been previously used as a dietary assessment method in other studies [16,17].

## Methods

### Subjects

From a previous health survey, researchers at the University of Aberdeen held a list of undergraduate students who had expressed interest in participating in future health surveys. This list was used to invite students via e-mail to take part in this study. This was supported by other methods including electronic noticeboard messages. Students were entered into a prize draw for shopping vouchers and were mailed a booklet of tailored diet feedback if they completed the study. Forty-one female and twelve male undergraduate participants completed four days of FoRC as well as four days food diary. Demographic data was collected for 52 participants. Students were living in university halls, in private accommodation or in the family home. Consent was presumed if participants approached the researcher to take part in the study after receiving the invitation.

### Confidentiality

No IRB approval was sought for this validation study. There were no confidentiality issues present in the study; participants were advised with the following statement: "All the information you provide is confidential". Personal information such as names and addresses were not released to anyone other than the first author, though these were kept for the purpose of individual feedback. Personal information was removed from the rest of the data and was stored separately where only the first author had access.

### FoRC

**Figure 1:**
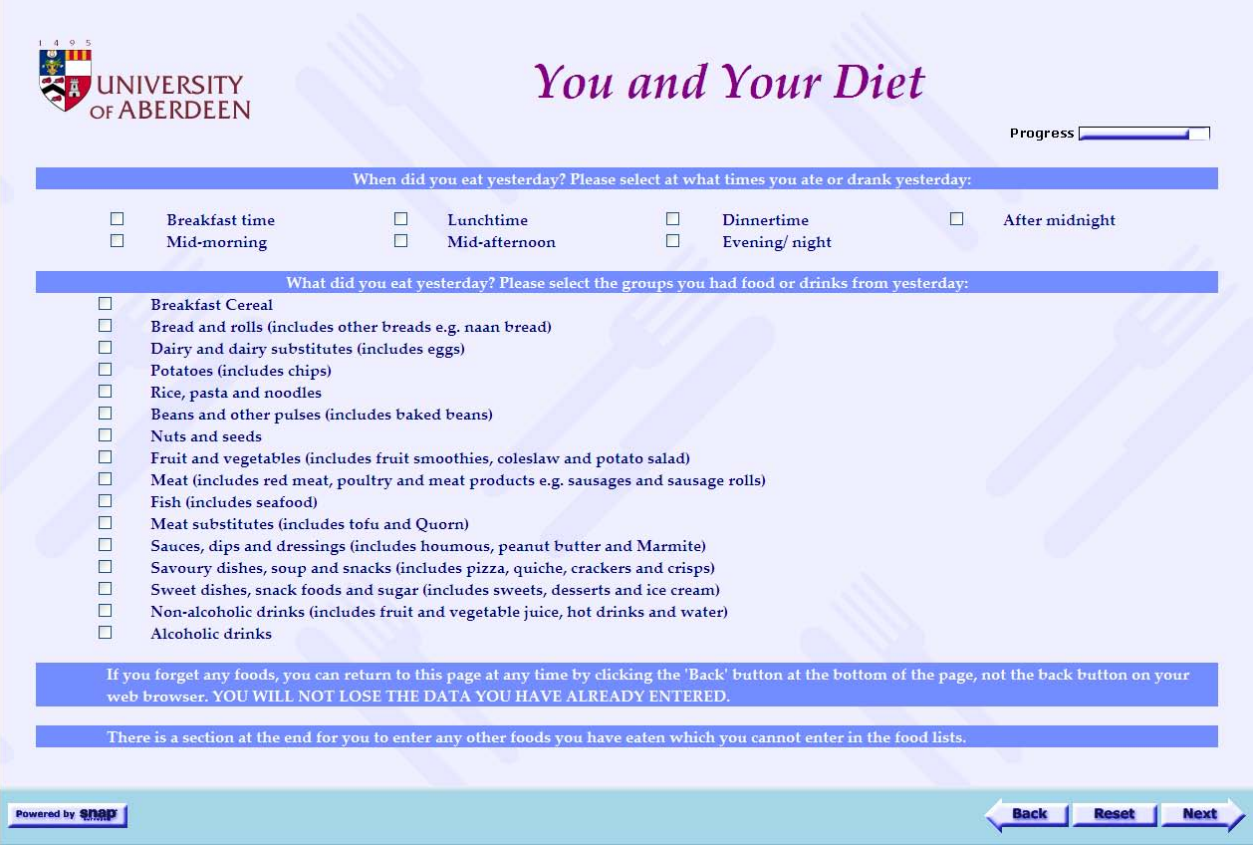
**First page of Food Recall Checklist (FoRC)**.

**Figure 2:**
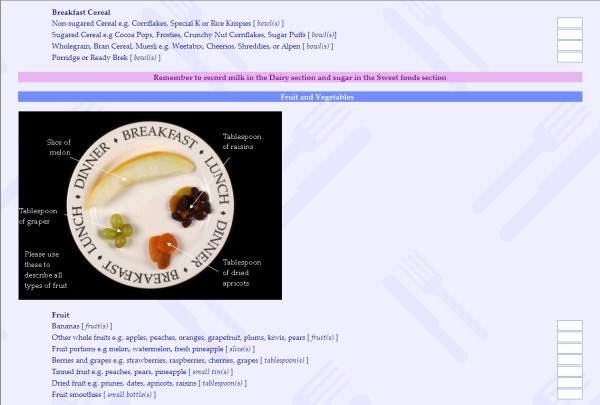
**Tailored food list in Food Recall Checklist (FoRC)**.

FoRC was developed to collect participants' dietary intake on the previous day via the Internet. The food list was developed with reference to existing dietary assessment methods [18] and more detailed investigations into the typical undergraduate diet. On the first page of FoRC, participants recorded the time periods when they consumed food and drink items on the previous day, then they selected the broad food and beverage groups consumed, choosing from a 16-item list (Figure 1). The next page showed a tailored list of the expanded elements for each of the main groups chosen on the previous page, accompanied by colour photographs of the selected foods (Figure 2). Inclusion of photographs in FoRC was designed to assist participants in estimating portion size [19]. The use of layers of questioning allowed participants extra time to recall foods and beverages consumed, as well as customizing the long food list to only the items participants had consumed.

In total, there were 121 food and drink items in FoRC, each of which included details of foods in the group, as well as a description of the intended portion size for each item. Participants recorded the number of portions of each food they consumed in a blank box at the end of the line. Pilot work had shown that students were more likely to complete four days of FoRC than seven days and that correlation between FoRC and the reference method was not drastically reduced when fewer days were collected (data not shown). Participants were asked to complete four consecutive days of FoRC (three weekdays and one weekend day). Instructions for completing the questionnaire were included online before participants began the questionnaire, but no further direction was given. Participants visited FoRC each day and recorded all foods and drinks consumed on the previous day. Participants also completed a demographic questionnaire and questions on their attitudes to food and healthy eating.

Online questionnaires, including FoRC were designed in Snap Survey Software v8 (SnapSurveys, Thornbury, Bristol, UK). Data collected online was imported into SPSS v14 (SPSS Inc, Chicago, IL, USA). Average daily nutrient intakes were calculated using data from UK food composition tables [20]. Each line in FoRC was assigned an average value for the nutrients of interest. The average value for each item in FoRC was calculated using values for commonly eaten foods in that category. Each item included foods which were similar in their content of the nutrients of interest. Around a quarter of FoRC entries required additional coding where participants had entered 'other foods'.

### Food diary

Following completion of four days of FoRC, participants completed a four-day non-weighed food diary (three weekdays and one weekend day), where foods and drinks were recorded as they were consumed. Pilot work had shown that a four-day non-weighed diary was highly correlated with a seven-day non-weighed diary for the foods and nutrients of interest (Spearman's rank correlation co-efficient 0.721–0.928, p ≤ 0.01) and that four days was completed by a greater number of participants.

Estimated food records have been previously used in comparison studies of dietary assessment methods [16,17]. The non-weighed food diary was chosen over the weighed food diary to reduce participant burden in a population who are often difficult to recruit and retain to dietary surveys. It was not known whether the food diary method would be agreeable to participants. FoRC was not compared to a traditional 24-h dietary recall as it was essential that the comparison method was not subject to the same errors of recall as the new method. A crossover design, where participants were randomly assigned to complete *either *FoRC *or *the diary first was not used, as it was more important that participants had no (recent) experience of completing any dietary assessment method.

Participants were asked to be specific in food descriptions in the four-day diary, using colour photographs included in the back of the diary as a guide to describe portion sizes (the same photographs were used in FoRC and the diaries). If foods were not adequately described, the participant was contacted for clarification, though this was only required in two cases. Weights of foods were estimated using known weights of the foods in the photographs. If photographs were not used as a reference by the participant, a weight was assigned to the item using the UK Food Portion Sizes guide [21] which provides information on typical weights and portion sizes of UK foods. Further information on portion or packet sizes was also researched at product or grocery shopping websites. Foods and beverages, along with estimated weights, were entered into WinDiets Research (N), diet analysis program (Univation Ltd, Aberdeen, UK) for calculation of average daily intakes. Each diary took the author up to forty-five minutes to code and to enter the data into WinDiets.

### Analysis

Male and female participants were analysed as one group due to the small sample size. Median intake and the 95% reference range were calculated for eight variables of interest: energy (kJ), fat (g), percentage food energy from fat, NSP (g), fruit and vegetables (g), breakfast cereal (g) and bread (g) and alcohol (units). Distribution of variables of interest was assessed using histograms and the one-sided K-S test for normality. Since not all intakes were distributed normally, the Spearman's rank correlation co-efficient was used to assess association between methods. The Wilcoxon signed ranks test was used to test whether variables differed significantly between the two measurements. As neither dietary assessment method is known to provide completely accurate data and strong association between methods does not imply agreement between methods, agreement was assessed using the Bland-Altman method [22]. Bland and Altman suggested that good *correlation *between methods occurs if points lie in a straight line, however points must lie along a line of equality for good *agreement *between methods. This method does not require Normal distribution of variables.

## Results

### Sample characteristics

542 students were contacted from the list of individuals interested in other health surveys, but only 34 of these individuals completed the study, a response rate of 6.3%. A further 19 students were recruited via the other recruitment methods, but response rate for the whole survey was incalculable as all students at the university were potentially exposed to the study. The population was aged 18–49 years and the mean age of the population was 23.2 (SD = 5.9) years. 73% of participants were aged 17–22 years and 92.9% of the sample was undergraduate, which implies the majority of the sample fitted the 'transition' group description. Where data was available, participant's self-reported weight and height were used to calculate BMI. BMI of the population ranged from 17–49 kg/m^2 ^and the mean BMI of the sample was 22.8 (SD = 5.0) kg/m^2^, which sits in the normal weight range.

### Dietary intake data

Table 1 (see Additional File 1) shows the median and 95% reference range for intakes of eight foods and nutrients from the diary and FoRC. Median energy intakes of 8185 kJ from the diary and 8007 kJ from FoRC were similar to what would be expected in a sample population with a mean BMI in the normal weight range [23]. According to the Wilcoxon signed ranks test, median energy (kJ) and alcohol (units) intakes were significantly lower in FoRC than in the diary. Median fruit and vegetable and breakfast cereal intakes were significantly higher in FoRC than in the diary. The diary showed a narrower range of measures than FoRC, except for percentage food energy from fat, bread (g) and alcohol intakes. Mean differences between methods were small, but there were wide limits of variation for foods and nutrients. Energy intake varied between methods by ± 5000 kJ and fat intake could vary by ± 60 g for a given individual. According to the mean differences, FoRC showed higher intakes than the diary for NSP (g), fruit and vegetables (g), bread and breakfast cereal (g). Table 1 (see Additional file 1) shows that correlation was generally high between the two methods. For all nutrients except alcohol, correlation co-efficients were statistically significant and were greater than 0.5 for all nutrients except percentage food energy from fat (0.33–0.76). This implied good agreement between the two methods for energy, fat, NSP, fruit and vegetables, bread and breakfast cereal, since correlation of greater than 0.5 is suggested as acceptable in a validation of a new dietary assessment method [24].

### Bland-Altman

**Figure 3:**
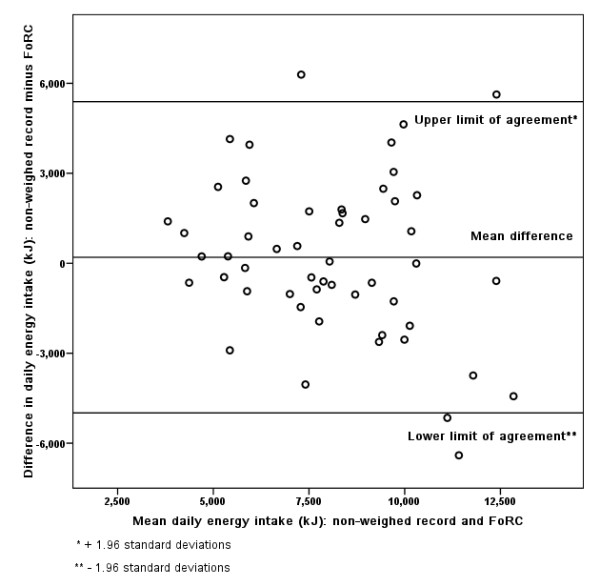
**Bland-Altman plot of energy intake (kJ): non-weighed record and FoRC**.

**Figure 4:**
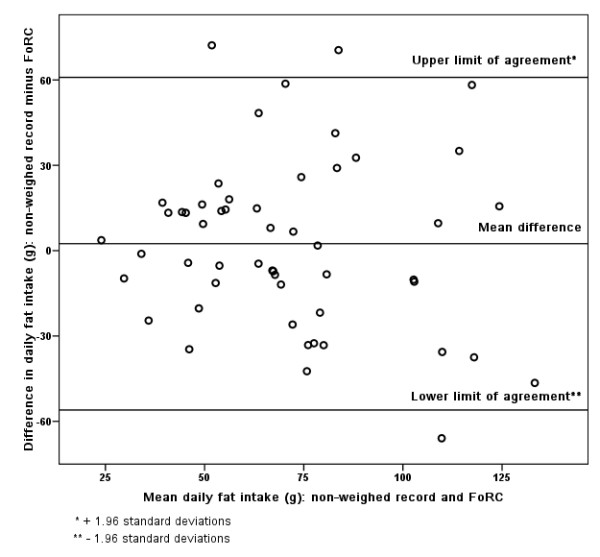
**Bland-Altman plot of fat intake (g): non-weighed record and FoRC**.

**Figure 5:**
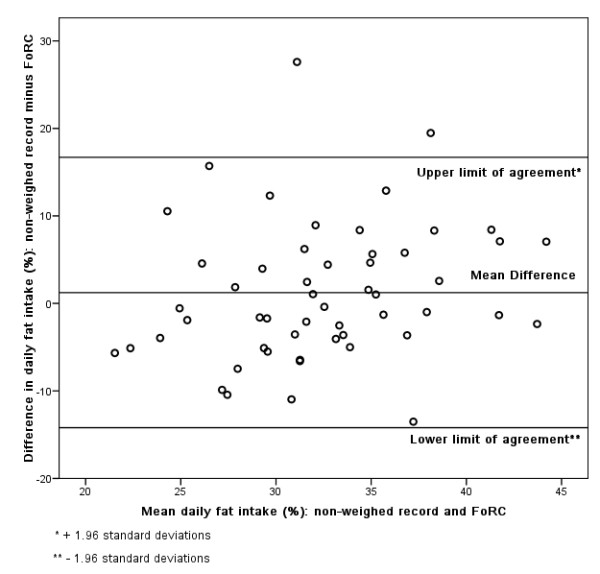
**Bland-Altman plot of fat intake (percent food energy): non-weighed record and FoRC**.

**Figure 6:**
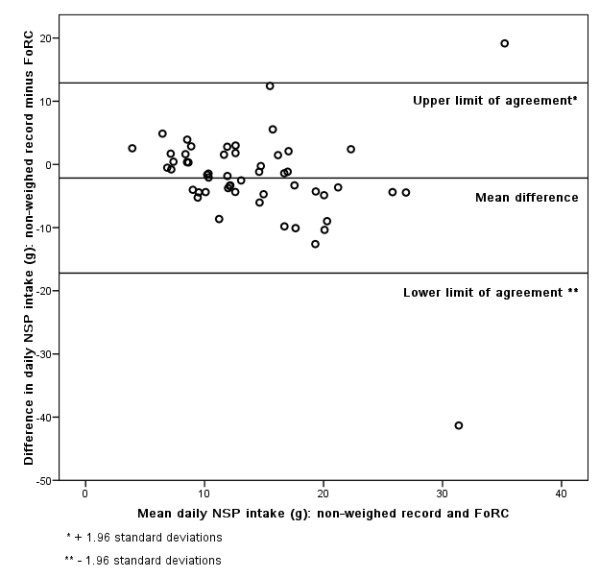
**Bland-Altman plot of NSP intake (g): non-weighed record and FoRC**.

**Figure 7:**
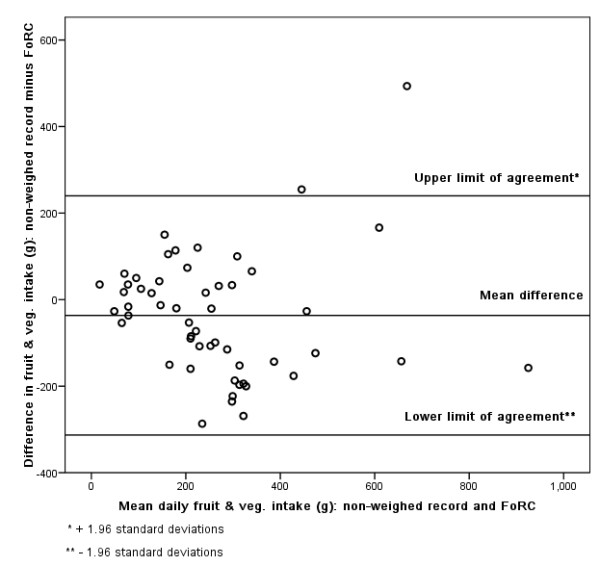
**Bland-Altman plot of fruit and vegetable intake (g): non-weighed record and FoRC**.

**Figure 8:**
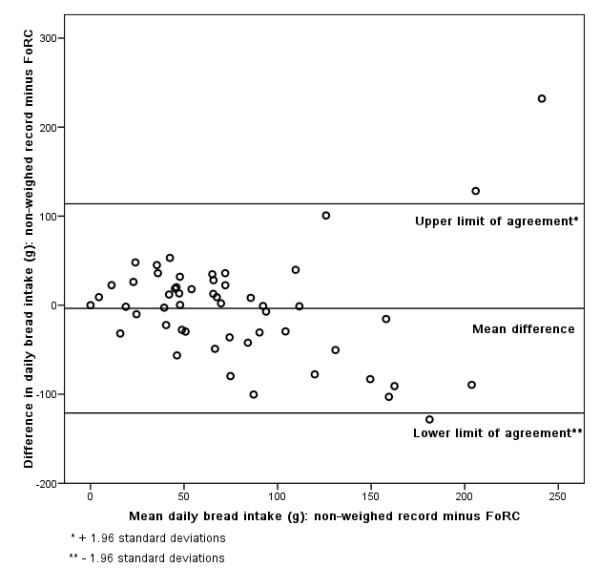
**Bland-Altman plot of bread intake (g): non-weighed record and FoRC**.

**Figure 9:**
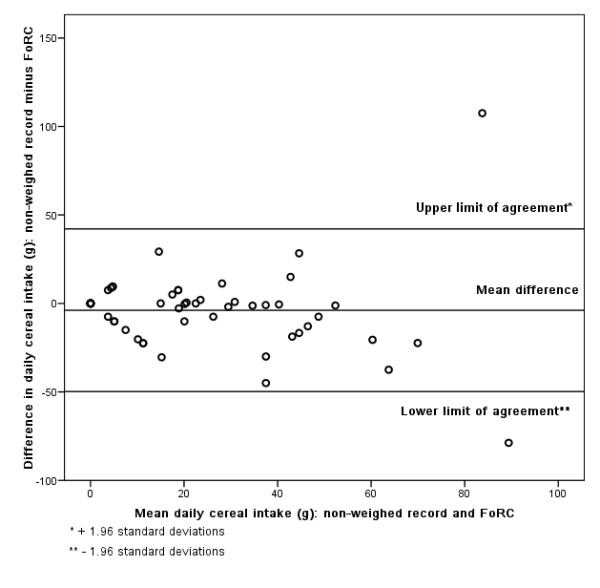
**Bland-Altman plot of breakfast cereal intake (g): non-weighed record and FoRC**.

**Figure 10:**
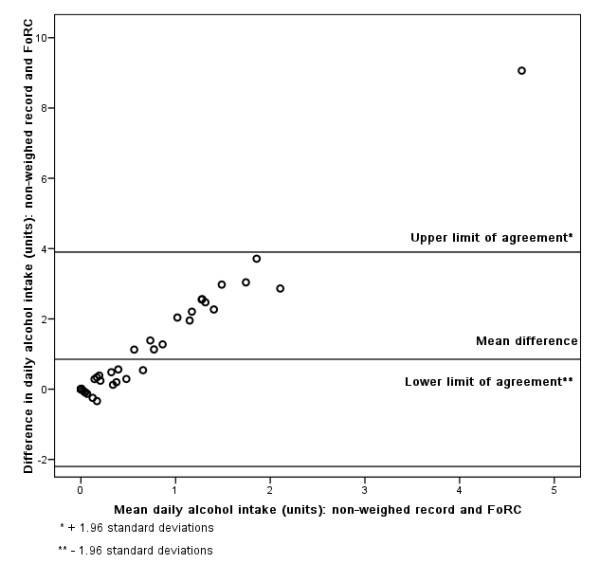
Bland-Altman plot of alcohol intake (units): non-weighed record and FoRC

Figures 3, 4, 5, 6, 7, 8, 9, 10 show the mean agreement and 95% confidence intervals between the diary and FoRC. Figure 3 shows mean energy intake from the diary compared to FoRC. At the absolute limits of variation, estimated energy intake could vary by up to 7000 kJ. In Figure 4, mean fat intakes (g) were widely spread and showed extensive variation. Figures 6, 7, 8, 9 and show Bland-Altman data for NSP (g), fruit and vegetables (g), bread (g) and breakfast cereal (g) intakes. The data were disrupted by a few large outliers in the each of the figures, but for the rest of the measures, results were concentrated around the mean difference line. However, for these nutrients and foods there was a tendency for wider differences between methods as the average intake from FoRC and the diary increased. There was no such pattern in the spread of data in Figures 3, 4, 5. Figure 10 shows the plot for alcohol and there was a clear indication that FoRC was more likely to underestimate alcohol intake the larger the amount of alcohol reported in the diary.

## Discussion

### Median intakes

In this study, application of the Wilcoxon Signed Rank test showed that median intakes of energy (kJ), fruit and vegetables (g), breakfast cereal (g) and alcohol (units) differed significantly from those reported in the diary. However, for most of the variables, median intakes from the two methods were extremely close. Perfect agreement between the two methods would not be expected since diet was only measured for 4 days by both methods, and it is likely that within-person variation in food and nutrient intake would have contributed to differences in intakes between the two methods

### Correlation

Correlation co-efficients between dietary assessment methods of greater than 0.5 for foods and 0.8 for alcohol have been suggested as acceptable in validation studies of FFQs and these have been applied here [24]. Due to lack of literature on cut-offs for a comparison study of repeated written 24-h recalls, FFQ cut-offs were applied here. Correlation was greater than 0.5 for all non-alcohol variables, except percentage food energy from fat, implying good correlation between the methods. Results for these six variables from FoRC were only as reliable as the original participant data entered into FoRC and provided an estimate of four days diet intake as a proxy for habitual dietary intake.

Correlation between methods of greater than 0.8 could be expected for alcohol [24], but correlation between methods in this study was well below this figure (r = 0.4). FoRC may not be particularly useful to capture alcohol intakes in this population. A young, undergraduate population may have unpredictable drinking patterns or may have had one 'peak' drinking event which was captured by one method and not the other and variation may be due to differences in behaviour, rather than the strength of one method over the other. Students may have been less inclined to use computers after drinking alcohol and may be unlikely to remember excessive alcohol consumption. Students may have been embarrassed to account for high alcohol intakes, which could also explain low median alcohol intakes reported in Table 1 (see Additional file 1). Highest alcohol intake over the measurement period may be a more useful measure.

Correlation co-efficients resulting from this study were deemed to be acceptable when employing cut-offs used in other dietary assessment comparison studies. Other dietary assessment comparison studies completed in young adult populations showed correlations ranging between 0.3 and 0.7 when results were reported for the same variables as this study [9,25-27]. None of the food and nutrients chosen or reference methods used in the other studies were exactly the same, but correlations from other studies show that results from this study were similar to or better than comparison studies of other new methods.

### Bland-Altman method

Bland and Altman state that their plot is intended to assess whether the variability of difference between measures is roughly constant across the range of measurements [28]. The small mean differences could mean that FoRC provides a reasonable estimate for group intakes of the eight variables of interest. There were wide limits of agreement for all foods and nutrients shown in Table 1 (see Additional file 1) and in Figures 3, 4, 5, 6, 7, 8, 9, 10, implying that individual results from FoRC should be interpreted with care. For NSP (g), fruit and vegetables (g), bread (g) and breakfast cereal (g), there was a tendency for wider differences between methods as the average intake from FoRC and the diary increased, but the data was not so widely spread as data for energy (kJ), fat (g) and percentage food energy from fat.

It is likely that there was less variation between FoRC data and the diary for NSP and fruit and vegetables, as well as for bread and breakfast cereal, as there were fewer subjective choices to be made by the researcher when the data was analysed. For example, 'bananas' were listed as a single item in FoRC, so the nutrient analysis of FoRC and the diary would be identical where a participant had consumed a banana. Conversely, if the participant had consumed a muffin, the analysis would not be the same for both methods, as this item would be accorded the average nutrient values for a 'cake' in FoRC, but would be analysed as a muffin in the diary. It may also be that FoRC reported higher intakes of fruit & vegetables and foods high in NSP because these are typically healthy choices which may be more likely to be recalled as they promote a more positive impression of the individual diet [29].

### Advantages of FoRC

Entering four-day diaries into a dietary analysis package was time-consuming. In comparison, FoRC took relatively little time to analyse. Average nutrient intakes could be calculated in SPSS for a large number of participants at the same time. Data collected in FoRC was returned precoded with nutrient values for each item consumed, whereas each item recorded in the food diary had to be coded before daily average nutrient intakes could be calculated. FoRC partially eliminated the issue of subjective judgements by researchers on which food code to assign to foods reported in FoRC as each item was precoded with nutrient values. The only area of responsibility for categorising foods eaten was where participants had included items in the 'other foods' section of FoRC. Print costs were over £6.00 per diary, whereas Snap Survey Software was accessed for free, using the university site licence. Money saved running FoRC online meant participants could be offered a prize draw incentive to take part.

Asking participants to complete records online removed the chance of researcher error when data was analysed in SPSS using the FoRC database. When unusual values were recognised in the FoRC analysis database during pilot work, these errors were rectified. FoRC was designed explicitly for the sample population, so the definitions and terms used should have help to avoid misclassification errors made by participants. Variability between the results from the two methods may have been in part due to natural variation in dietary intake during the two measurement periods and may not be a reflection of the poor performance of one method in comparison with the other.

### Limitations

The number of responses to this study was low. An accurate response rate could not be calculated, because it was unclear how many individuals were exposed to recruitment methods in the study. The response rate of 6.3% from students previously exposed to another health survey highlights the difficulties of recruitment, even in participants who have indicated they are interested in participating in health surveys. However, in other dietary studies in populations of similar age and education, numbers of respondents were also low [9,27].

The sample in this study was unlikely to be representative of the general student population, given that it required eight days participation and could not offer an incentive to every participant. Recruiting individuals from another health survey meant that students who took part were more likely to be interested in health-related matters than the general student population. A large number of students may have been left out of the recruitment process and students fatigued with other studies may have been discouraged from taking part. It is also likely that students with a prior interest in healthy eating would have been more motivated to take part and giving diet feedback as an incentive may have increased 'healthy subject' bias in the sample. The sample was not representative of male students at the university since 79% of the sample was female. However, it was likely that the study took place in a sample representative of any future study populations who would complete FoRC. It is possible that taking part in any dietary assessment survey may encourage participants to alter their food intake and this may affect whether the results are a true reflection of *habitual *dietary intake.

The author was responsible for the calculation of the nutrient values to analyse FoRC as well as the food diary analysis, so it is possible that there was greater matching of food items from the diary to similar items in FoRC, increasing agreement between methods. However, apart from 'other foods' recorded, data returned from FoRC was precoded, so it was not possible or the author to change food code allocation after information was entered by participants. This issue was also potentially less damaging than risking inter-researcher variation in diary analysis. Since dietary intake data is subjective for participants and researchers, assumptions about food choices were made at each stage in the data entry and analysis, though FoRC was designed with photographs, examples and portion guides, which should have removed some participant subjectivity. However, if participants were unsure of where foods they had consumed should be recorded in the checklist they may have selected the incorrect category, instead of using the 'other foods' section.

It is not known how accurately participants adhered to the study protocol. For example, it is possible that diaries were completed at the end of the day, rather than throughout the day and that FoRC may have been completed at the end of the day the record was about, rather than on the following day. Although the diary was not subject to the same errors of recall as FoRC, errors in portion size estimation by participants may have been similar in both methods due to the use of food photographs for portion size estimation in both methods.

Errors in assigning nutrient values to foods in the FoRC database and the diaries could have occurred as the UK food composition tables are not complete. It was not possible to avoid errors introduced by the participants in FoRC, e.g. the participants may have entered the number of portions consumed into the wrong box in FoRC, which would have resulted in an incorrect estimate of their dietary intake. Data entered in FoRC was subject to errors of recall. The same photographs were used in FoRC and the diaries, so good correlation between the two methods may in part be due to participants becoming accustomed to the photographs. New colour photographs were used in both methods, instead of photographs from a food atlas [30]. It would not have made sense to use different photographs between the two methods and using the same photographs in each method should not affect the results of the study.

The Snap Survey Software questionnaire design package did not have the capacity for certain design aspects. FoRC could have greater capacity to prompt participants for forgotten foods, in a manner similar to the Automated Multiple Pass Method [31], but at this time it was limited by the design software. However, making FoRC more complicated to complete might be off-putting for potential respondents and increasing the amount of data collected in FoRC would make data analysis more complex and time-consuming. The comparison made between the two methods was four days diet from each record, so it was unlikely that methods would agree exactly and there was a large chance that neither record was representative of the habitual diet of students.

## Conclusion

Data from four days of FoRC was shown to have high (r > 0.5) correlation with four days non-weighed food diary for energy (kJ), fat (g), NSP (g), fruit and vegetables (g), bread (g) and breakfast cereal (g). FoRC was quicker and more cost-effective to implement and analyse than a food diary and took advantage of a novel way to assess diet. FoRC could be used in place of a diary in this sample. Correlation between FoRC and the reference method was often as good as or better than correlations found in other studies, though comparison was limited due to lack of similarity between the studies. Although variation within individuals was wide when FoRC was compared with a four-day non-weighed food diary, group results from FoRC could be useful. Individual results from FoRC may not be used to recommend precise dietary changes. However, if used with appropriate caution, results from FoRC may be used to tailor more general dietary messages towards individuals.

Comparison studies of dietary assessment methods may be difficult to implement due to lack of interest in dietary surveys amongst students, but FoRC was shown to compare favourably to the reference method in terms of convenience, cost-effectiveness and avoiding data-entry errors. Memory errors and misclassification of foods by participants may have occurred in the FoRC dietary assessment method, but the advantages of the method were believed to outweigh the risk of these errors. FoRC was therefore found to be an appropriate dietary assessment method for assessing diet in an undergraduate student population.

### Future work

There are a number of improvements which could be made to the existing FoRC questionnaire. For example, advances in questionnaire software could allow participants to be prompted for food items which are likely to be consumed together where one entry might remind participants about another such as bread and butter, cereal and milk, coffee and sugar. Participants could also be consulted on ways to improve the questionnaire. The next steps are to develop healthy diet feedback messages tailored to data collected in FoRC which could be tested for effectiveness in an undergraduate student population.

## Competing interests

The authors declare that they have no competing interests.

## Authors' contributions

FC completed questionnaire design, recruitment, data collection and analysis and drafted the manuscript. GM provided advice on study design and interpretation of results. LM provided advice regarding interpretation and presentation of results. All authors read and approved the final manuscript.

## Supplementary Material

Additional file 1 **Table 1.** Median and range of daily intakes of eight nutrients and foods from the non-weighed record and FoRC with measures of agreement between methods. Click here for file
