# Supplementary material for: A novel online Food Recall Checklist for use in an undergraduate student population: a comparison with diet diaries
Source: Nutr J. 2009 Feb 19;8:13. doi: 10.1186/1475-2891-8-13 (PMC2654910; doi:10.1186/1475-2891-8-13)
Supplement: Additional file 1 — Table 1. Median and range of daily intakes of eight nutrients and foods from the non-weighed record and FoRC with measures of agreement between methods. [file 1475-2891-8-13-S1.doc]

**Table 1** Median and range of daily intakes of eight nutrients and foods from the non-weighed record and FoRC with measures of agreement between methods

|  |  |  |  |  |  |  |  |  |  |  |  |  |  |
| --- | --- | --- | --- | --- | --- | --- | --- | --- | --- | --- | --- | --- | --- |
|  |  | **Non-weighed record** | |  | **FoRC** | |  | **Difference between methods** | | | |  | **Correlation** |
|  |  |  |  |  |  |  |  |  |  |  |  |  |  |
|  |  |  |  |  |  |  |  |  |  |  |  |  |  |
|  |  | **Median** | **Range^a^** |  | **Median** | **Range^a^** |  | **Mean^b^** | **SD** | **Lower limit^c^** | **Upper limit^d^** |  | **r** |
|  |  |  |  |  |  |  |  |  |  |  |  |  |  |
|  |  |  |  |  |  |  |  |  |  |  |  |  |  |
| ***Energy (kJ)*** |  | 8185.0 | 9737.5 |  | 8007.0** | 11705.4 |  | 199.5 | 2647.5 | -4989.6 | 5388.5 |  | 0.58^++^ |
|  |  |  |  |  |  |  |  |  |  |  |  |  |  |
| ***Fat (g)*** |  | 63.7 | 115.1 |  | 63.5 | 133.7 |  | 2.5 | 29.8 | -56.0 | 60.9 |  | 0.53^++^ |
|  |  |  |  |  |  |  |  |  |  |  |  |  |  |
| ***Fat (% food energy)*** |  | 32.3 | 28.6 |  | 32.3 | 26.8 |  | 1.2 | 7.9 | -14.2 | 16.7 |  | 0.30^+^ |
|  |  |  |  |  |  |  |  |  |  |  |  |  |  |
| ***NSP (g)*** |  | 11.4 | 28.6 |  | 13.8 | 40.9 |  | -2.2 | 7.7 | -17.2 | 12.9 |  | 0.68^++^ |
|  |  |  |  |  |  |  |  |  |  |  |  |  |  |
| ***Fruit and veg. (g)*** |  | 198.8 | 863.1 |  | 258.8* | 893.2 |  | -36.6 | 140.8 | -313.0 | 239.9 |  | 0.70^++^ |
|  |  |  |  |  |  |  |  |  |  |  |  |  |  |
| ***Bread (g)*** |  | 69.0 | 313.6 |  | 63.0 | 247.4 |  | -3.6 | 60.0 | -121.1 | 113.9 |  | 0.76^++^ |
|  |  |  |  |  |  |  |  |  |  |  |  |  |  |
| ***Breakfast cereal (g)*** |  | 20.0 | 98.1 |  | 22.5* | 112.6 |  | -3.9 | 23.4 | -49.8 | 42.1 |  | 0.69^++^ |
|  |  |  |  |  |  |  |  |  |  |  |  |  |  |
| ***Alcohol (units)*** |  | 0.3 | 12.2 |  | 0.0** | 4.6 |  | 0.9 | 1.6 | -2.2 | 3.9 |  | 0.40^++^ |
|  |  |  |  |  |  |  |  |  |  |  |  |  |  |

^a^ 95% reference range around the median

^b^ Mean difference = mean daily diary intake- mean daily FoRC intake for each indvidual

^c^ Mean difference - 1.96*SD

^d^ Mean difference + 1.96*SD

* Median intake from FoRC was significantly different to median intake from non-weighed record at the 0.05 level

** Median intake from FoRC was significantly different to median intake from non-weighed record at the 0.01 level

^+^ Spearman’s rank correlation co-efficient was significant at the 0.05 level

^++^ Spearman’s rank correlation co-efficient was significant at the 0.01 level
